# Healthy Weigh (*El camino saludable*) Phase 1: A Retrospective Critical Examination of Program Evaluation

**Published:** 2006-06-15

**Authors:** Pamela Jean Frable, Lyn Dart, Patricia J Bradley

**Affiliations:** Harris School of Nursing, Texas Christian University; Department of Nutritional Sciences, Texas Christian University, Fort Worth, Tex; Harris School of Nursing, Texas Christian University, Fort Worth, Tex

## Abstract

**Background:**

Healthy Weigh (*El camino saludable*) is an obesity prevention program for low-income, predominantly Hispanic and African American families in an urban community in Tarrant County, Texas. Healthy Weigh Phase 1 was a successful community–campus partnership that took place in summer (June–August) and fall (September–November) 2003. The program met stated objectives and extensively engaged students from several health disciplines in service learning. This article describes what we learned about the evaluation of the program by examining the phase 1 evaluation process.

**Context:**

Family environments are important intervention settings for establishing life-long dietary practices. Available in English and Spanish, Healthy Weigh Phase 1 helped families that were at risk for overweight and obesity to adopt healthy eating, physical activity, and weight management patterns.

**Methods:**

Analysis of a program logic model and formative evaluation data identified evaluation questions that could have improved the phase 1 evaluation process. Questions were categorized according to Donabedian's structure–process–outcome framework, and potential benefits of each question were identified. The Centers for Disease Control and Prevention's *Framework for Program Evaluation in Public Health *standards were used to judge the overall quality of the phase 1 evaluation process.

**Consequences:**

The phase 1 evaluation process successfully assessed the program's effects and generally met evaluation standards. Our critical examination also highlighted structure and process evaluation issues with potential for strengthening future interventions, community partnerships, and program outcomes.

**Interpretation:**

Lessons learned influenced the phase 2 grant activities. Most importantly, we learned that involvement of program participants as full partners in program design, evaluation, and implementation is essential. Our understanding and practice of program evaluation evolved as Healthy Weigh became a true community-based participatory research endeavor.

## Background

Weight management requires a complex series of interactions of individual choices, behaviors, and environments (e.g., familial, socioeconomic, political, cultural, natural, built [manmade]) (1). Healthy Weigh (*El camino saludable*) is a community-based obesity prevention program for low-income, predominantly Hispanic and African American families in an urban community in Tarrant County, Texas. Phase 2 has already been completed, and in spring 2005, Healthy Weigh implemented post-phase 2 activities (sidebar). Healthy Weigh Phase 1, a community–campus (Texas Christian University, Fort Worth, Tex) partnership, was completed in summer (June–August) and fall (September–November) 2003. The program met stated program objectives and extensively engaged students from several health disciplines in service learning (2). As phase 1 concluded, Healthy Weigh evolved from research being conducted in a community to community-based participatory research. This article describes lessons learned from critically examining the phase 1 evaluation process.

**Table:** 

**Healthy Weigh (*El camino saludable)* Program Overview**
Child care for preschool-aged children, free family food bags, and transportation were provided in each phase. Services were offered in English and Spanish.
**Phase 1 (Summer and Fall 2003)**
Age-, gender-, and ability-appropriate physical activity classes
Family meals with table talks
Nutrition lessons for adults, adolescents, and school-aged children
**Phase 2 Pilot (Spring 2004)**
Physical activity classes and a healthy snack with an age-appropriate nutrition lesson
**Phase 2 (Fall 2004)**
Family exercise and nutrition classes
Light, healthy family meals with nutrition and food demonstrations
**Post-Phase 2 Exercise Program (Spring 2005)**
Age-appropriate physical activity classes and a healthy family snack
**Family Cooking Course (Spring 2005)**
Series of four cooking classes in which families worked together to prepare nutritious meals

Program evaluation is an essential part of community health practice and has four goals: to gain insight, change practice, assess effects, and affect people participating in the evaluation process (3). The request for proposals, grant preparation, and implementation of phase 1 occurred rapidly. The grant's purpose, which was to reduce overweight and obesity, required a program evaluation focused on assessing effects. However, in this pilot intervention, we also wanted to gain insight about the most effective ways to help low-income, racially and ethnically diverse families adopt healthy eating and physical activity patterns and manage their weight. Formative evaluation suggested that adding questions to the phase 1 evaluation process could have improved the program's efficiency and effectiveness and provided more insight into interactions among the program, participants, and community environments. We critically examined the phase 1 evaluation process using the formative evaluation data, Donabedian's structure–process–outcome framework (4), and the Centers for Disease Control and Prevention's (CDC's) *Framework for Program Evaluation in Public Health* (referred to as *framework *in this article) standards (3). This examination provided information to improve delivery and evaluation of subsequent Healthy Weigh interventions.

## Context

### Overweight and obesity as community health problems

The prevalence of overweight and obesity in Texas is among the highest in the United States, where overweight and obesity rates have increased steadily in the past two decades (5-7). Sixty-four percent of adult Texans are overweight or obese, and more than 39% of fourth-graders, 37% of eighth-graders, and 29% of eleventh-graders are overweight or at risk for overweight (5). Obesity is more prevalent among women, African Americans, Hispanic Americans, and people with low education levels and income. Many racial and ethnic groups continue to face disproportionate risks for obesity-related chronic diseases and associated decreased life expectancy (8,9).

Recent speculation attributes the increasing obesity prevalence among low-income populations to socioeconomic status, personal choices, established cultural and family patterns, and environmental factors (10-13). Low-income communities are particularly vulnerable because nutritious foods can be expensive or difficult to find, whereas less healthy, energy-dense foods are readily accessible and affordable (11). Compared with higher income neighborhoods, poor neighborhoods may lack well-maintained sidewalks and streets, safe outdoor spaces, or exercise facilities and therefore be less conducive to physical activity (14).

Parents are primary sources of information for children who are learning about healthy eating practices, so family environments are important intervention settings for establishing lifelong dietary habits (14-16). Studies investigating familial aggregation of obesity show that family eating environments link parental adiposity and dietary intake with children's adiposity and dietary intake (17). 

### The Healthy Weigh (*El camino saludable*) program

A request for proposals to address overweight and obesity from United Way of Metropolitan Tarrant County prompted university faculty and community organizations to collaborate on designing and implementing Healthy Weigh. As phase 1 was ending, development of a participant leadership group led to more balanced partnerships among participants, community organizations, and investigators, and Healthy Weigh evolved into a community-based participatory research effort. Texas Christian University Human Subjects Institutional Review Board approved the Healthy Weigh program. The previous sidebar summarizes the Healthy Weigh interventions; participants helped design and evaluate phase 2.

### Community characteristics

Cornerstone Community Center (CCC), a faith-based community organization, provided the physical facility for Healthy Weigh, assisted with weekly reminder calls to participants, and was the primary source of participant referrals and community volunteers. CCC serves a community at high risk for overweight and obesity. Factors for overweight and obesity included the median household income ($20,000), federal poverty level (44.6% below the poverty level), and people older than 25 years lacking high school diplomas (42%) (18). The population in CCC's catchment area is 35% African American and 26% Hispanic (18). 

### Healthy Weigh Phase 1

Phase 1 was offered twice in 2003 (summer and fall) and consisted of 12 weekly sessions, prescreening, and postscreening. Participants chose to enroll in the English- or Spanish-language version of phase 1. Community focus groups provided input on program design, evaluation, and recruitment. Recruitment flyers were distributed door-to-door in target neighborhoods and through schools and CCC.

Of 282 people screened for phase 1, most participants were female (72%) and Hispanic (82%) or African American (12%). Many of the participants were families; 46% of participants were younger than 19 years,  and 9% were older than 60 years. Many participants had limited proficiency in English or Spanish. Based on body mass index, 84% of adults were overweight or obese, and 50% of children and adolescents were overweight or at risk for overweight.

The research design included evaluation of program process objectives and participant outcome objectives based on *Healthy People 2010* (1), *Recommendations to Increase Physical Activity in Communities* (19), and *Dietary Guidelines for Americans* (20). Following are the outcome objectives (21) and program process objectives for Healthy Weigh Phase 1:

**Outcome objectives**

1. Participants will demonstrate a statistically significant improvement in nutrition and exercise knowledge scores.
2. Seventy percent of participants 5 years or older will report dietary improvements from baseline to model component of the 2000 *Dietary Guidelines for Americans*.
3. Seventy percent of participants will report an increase from self-reported baseline in frequency, intensity, or duration of exercise.
4. Fifty percent of the adolescent and adult participants who are overweight at baseline will show an improvement in weight management.
5. Seventy percent of adult participants completing the program will achieve their individually determined nutrition or activity objective.

**Process objectives**

1. Sixty percent of the participants will complete the program by attending at least 8 of the 12 sessions and providing follow-up data.
2. Seventy percent of the adult and teen participants will report "good" or "very good" levels of satisfaction with the program.
3. Seventy percent of the children will report satisfaction with the food served and education sessions.
4. At the end of the program, 70% of participants who were overweight at baseline will write a goal for the next 12 weeks for their end-of-program weight management progress.
5. At the end of the program, 70% of participants will write an activity goal, dietary goal, or both for the next 12 weeks for their individually determined nutrition and exercise plans.
6. The Cornerstone Health Action Group will be formed and will establish contact with the Tarrant Area Food Bank, Fort Worth Public Health Department, and Texas Cooperative Extension.

Program evaluation was determined by interview, direct measurement, and self-report (Table 1).

The university research team (two registered nurses and one registered dietitian) directed program implementation, which was carried out by registered nurses, a registered dietitian, paid staff, community volunteers, and supervised students from nursing, nutritional sciences, kinesiology, social work, and medicine programs. More than 160 undergraduate and graduate students from two universities served as educators, exercise leaders, meal preparation and service coordinators, table-talk leaders, child care workers, health screeners, and research assistants. Sixty percent of the students were volunteers, and most of the remaining students participated to meet course requirements. Many program participants also served as volunteers by helping with set up, clean up, and interpretation.

## Methods

We analyzed evaluation data and a logic model (Figure) based on actual implementation of phase 1 (3). We used the analysis to identify evaluation questions that were missing from the phase 1 evaluation process. We categorized these questions as *structure, process,* and *outcome* and determined how including these questions in the phase 1 evaluation might have strengthened program implementation and outcomes (Tables 2, 3, and 4). Finally, we applied the following 30 framework standards to judge overall quality of the phase 1 evaluation process (3):

**Utility standards:** Does the evaluation serve the information needs of the intended users?

- Stakeholder identification
- Evaluator credibility
- Information scope and selection
- Values identification
- Report clarity
- Report timeliness and dissemination
- Evaluation impact

**Feasibility standards:** Is the evaluation realistic, prudent, diplomatic, and frugal?

- Practical procedures
- Political viability
- Cost-effectiveness

**Propriety standards:** Is the evaluation legal, ethical, and considerate of the welfare of those involved and affected?

- Service orientation
- Formal agreements
- Rights of human subjects
- Human interactions
- Complete and fair assessment
- Disclosure of findings
- Conflict of interest
- Fiscal responsibility

**Accuracy standards:** Does the evaluation reveal and convey technically accurate information?

- Program documentation
- Context analysis
- Described purposes and procedures
- Defensible information sources
- Valid information
- Reliable information
- Systematic information
- Analysis of quantitative information
- Analysis of qualitative information
- Justified conclusions
- Impartial reporting
- Meta-evaluation

**Figure 1:**
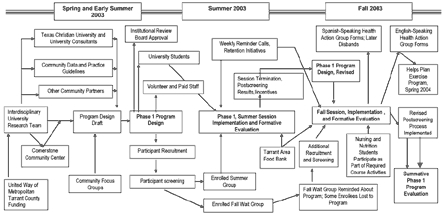
Healthy Weigh *(El camino saludable)* Phase 1 logic model.

## Consequences

### Utility standards

Seven framework utility standards address information needs of evaluation users, who were defined as the funding agency and investigators in phase 1. Ways in which utility standards were met included the following:

- The evaluation involved a credible interdisciplinary investigation team.
- The process and outcome objectives that were evaluated addressed information needs stipulated in the grant proposal.
- The methods for evaluating the outcome objectives were consistent with standard guidelines and values.
- Timely evaluation reports presented results in plain language (21).
- The funding agency's unsolicited award of a second year of funding confirmed the evaluation's impact.

There were several ways in which the evaluation could have been improved to meet utility standards (Table 2). Because other community stakeholders (CCC, program participants, and staff) were not identified as evaluation users and did not help design the evaluation plan, their information needs were unknown and not addressed. Monitoring the balance of needs is essential to developing effective community-based participatory research partnerships (22). Establishing evaluation criteria with partners could have promoted ongoing monitoring of relationships, frequent acknowledgment of strengths and growth, and rapid response to actual and potential problems.

### Feasibility standards

Three framework feasibility standards were developed to ensure effective and practical use of program resources. Most phase 1 evaluation procedures were practical, adapted for the community setting, and politically viable, and they effectively used time and resources. Use of familiar equipment (e.g., scales, stadiometer, measuring tape, sphygmomanometer) and noninvasive methods for data collection fostered participant acceptance of measurements and made it easier for students to help conduct screenings. Concerted efforts to prevent phase 1 activities from interfering with CCC's daily operations and early negotiation for using equipment and space helped gain and sustain CCC's cooperation in evaluation procedures. Proactive communication with CCC's staff and university, funding agency, and community–partner organizations also contributed to political viability. Ongoing budget management based on financial requirements of the program, university, and funding partner helped ensure that resources were used effectively to achieve program processes and outcomes.

Structure–process–outcome analysis highlighted ways the evaluation process could have better met the feasibility standards related to use of resources and political viability (Tables 2–4). Investigators frequently filled staff roles because of a shortage of staff members, lack of availability of staff members, or staff members' lack of skills. Resolving staff structure issues would have permitted more consistent staff assignments and potentially increased skills and job satisfaction. Greater attention to staff structure would have contributed to more efficient and effective use of resources for participant screening and overall program administration.

Two process variables also affected feasibility. Knowing more about why participants attended some sessions and not others could have promoted more cost-effective use of human and material resources needed for program physical activity and nutrition classes, family meals, and food-bag preparation. Systematically collecting data about participants who dropped out of the program might have helped improve retention and program design. Early clarification and monitoring of community partners' roles would have provided a framework for increased collaboration and communication among partners, making the evaluation process more politically viable.

### Propriety standards

Eight framework propriety standards address legal and ethical characteristics and how the evaluation process affects all the participants involved. Although the lack of formal agreements among all key parties involved in the evaluation was a weakness, overall the evaluation process met propriety standards. Concern for participant welfare was evident in design of evaluation tools and procedures, the willingness to adapt the research design to meet participant needs, the commitment to building relationships with participants and CCC staff, and adherence to financial accounting standards.

Informed consent and other screening tools were appropriate for participants with limited English proficiency. Documents were available in English and Spanish and were prepared at the fourth- to fifth-grade reading level in English, with reader-friendly formatting and adequate white space (23). Because one consent form per family was used, parents of large families and people with limited literacy did not have to handle multiple documents. Family members could choose to read the informed consent or have it read to them. Child care for preschool-aged children allowed families to concentrate on completing the informed consent.  

Participant privacy was maintained at levels consistent with community expectations. Staff members explained screening results and gave families copies of results in appropriate languages. Referrals for follow-up health care were based on prescreening findings. Efforts were made to obtain health care for participants who lacked regular sources of care.

When formative evaluation showed that participants were unfamiliar with setting goals, we modified the screening protocol to include teaching participants about setting appropriate goals for healthy eating, physical activity, or weight management. Fifty summer participants asked to enroll in the fall program, so we revised the research design. Relationships with participants and their progress toward improved physical activity and healthy eating patterns were more important to overall program goals than strict adherence to the original research design.

All financial reporting requirements of the university and United Way were met. Maintaining and properly cleaning the CCC facility, equipment, and supplies during and after each Healthy Weigh meeting also addressed fiscal responsibility standards.

### Accuracy standards

Twelve framework accuracy standards assess correctness of evaluation findings. The evaluation process for phase 1 grant objectives (see previous lists of outcome and process objectives) met these standards.

Structure–process–outcome analysis revealed how the phase 1 evaluation process could have measured the findings more completely and accurately (Tables 2–4). Collection of qualitative data was not as consistent and systematic as planned because of human resource problems. For example, although many table-talk conversations were recorded in log books, different staff members facilitated and documented the talks each week. Some weeks, too few staff members were available to document all talks, or available staff members lacked skills to document Spanish-language table talks. These factors contributed to uneven and inconsistent qualitative data collection. Most questions posed for improving the phase 1 evaluation process could have been answered by consistent, systematic collection and analysis of field observations, personal narratives, and table-talk conversations. Systematic identification, documentation, and analysis of best practices would have accomplished the following:

- Provided clear, complete understanding of effective participant recruitment and retention strategies
- Increased opportunities to build student–participant relationships
- Promoted investment of resources in staff building rather than managing staff turnover and shortages 
- Produced more comprehensive assessments of service learning in this program

## Interpretation

We undertook this critical examination of the Healthy Weigh Phase 1 evaluation to improve the evaluation design for future Healthy Weigh interventions. We learned that the phase 1 evaluation process successfully assessed program effects and generally met framework standards. Structure–process–outcome analysis highlighted additional evaluation factors related to structure and process that could strengthen subsequent Healthy Weigh interventions, community partnerships, and program outcomes.

We have already applied lessons learned to improve the phase 2 evaluation plan and process. An English-speaking health action group formed during phase 1 helped design the phase 2 pilot. Although this particular health action group disbanded, participants and students who had been consistently active in Healthy Weigh formed a new health action group. This group of monolingual English speakers, monolingual Spanish speakers, bilingual speakers (English and Spanish), adults, and youths acted as a true partner in the design, implementation, and evaluation of phase 2.

Another improvement was increased attention to monitoring the CCC–university partnership. Problems were avoided or addressed early in phase 2 by more frequent and intentional monitoring of the partnership. Contacting CCC after each Healthy Weigh meeting helped us be aware of and address actual or potential problems.

This critical examination was valuable and challenging. As is frequently true of community health programs, the opportunity and resources to address a significant community health issue emerged rapidly. Faced with limited development time, we focused on creating a phase 1 evaluation plan that would assess effects of an evidence-based program and would be designed to be culturally and linguistically appropriate for the target community. Recognizing Healthy Weigh as a pilot intervention, we wanted to gain insights about factors and relationships that would optimize community partnerships, program design, service learning, and improvements in participant families' physical activity, healthy eating, and weight management behaviors. Formative evaluation data gathered during program implementation suggested that the investigators were frequently filling volunteer and paid staff roles and not obtaining all desired data.

Critical examination of phase 1 evaluation enabled us to identify structure and process evaluation questions to include in future evaluation plans. These questions will enhance our insight and strengthen programs and their evaluation processes. Healthy Weigh has evolved into a community-based participatory research intervention as community partnerships have been established and nurtured. This retrospective examination made clear that we began Healthy Weigh Phase 1 without the community relationships necessary to involve all stakeholders in the evaluation process as we now envision and practice it.

## Figures and Tables

**Table 1:** Data Collected for Program Evaluation of Healthy Weigh Phase 1, Tarrant County, Texas, 2003

**Objective Type**	**Instrument**	**Data Collected**
Outcome objectives	Health profile questionnaire	Demographics, health history, physical activity patterns
	Health screening physical assessment	Blood pressure, body composition measures
	Nutrition and exercise knowledge quiz	Nutrition, exercise, and weight management knowledge
	Food frequency questionnaire 24-hour diet recall	Dietary practices and eating patterns
Process objectives	Attendance records	Participant weekly attendance
	Table-talk logs	Content of table-talk dialogues
	Program satisfaction survey	Participant feedback on Healthy Weigh Phase 1
	Participant goal sheet	Nutrition goals, exercise goals, weight-management goals, or all of these
	Health action group meeting minutes and attendance record	Health action group development, discussions, and participation

**Table 2:** Structure Evaluation^a^ Questions and Potential Benefits for Healthy Weigh Phase 1, Tarrant County, Texas, 2003

**Structure Question**	**Potential Benefits for Healthy Weigh Phase 1**
Was the staff adequate in numbers, hours available, and skills? Volunteer staff Paid staff	Investigators would be able to spend more time coordinating the research and administering the overall program instead of filling paid and volunteer staff roles. Consistent volunteer and paid staff assignments would allow volunteer and paid staff to improve their skills and gain greater satisfaction from their participation. Consistent documentation of table talks, personal narrative, and field note data could occur.
Was the facility appropriate and adequate for the program? Host facility Back-up facilities	Intentional evaluation of facilities could create opportunities to include funding for facility repairs and updates in future Healthy Weigh budgets.
Were material resources adequate in quantity and type to conduct the program? Physical activity classes Nutrition classes Meal preparation and service Child care Screenings Administration	Identifying efficient and effective strategies can help manage program components affected by limited resources.
Were community partners and their roles clearly identified before the program began?	Clarification of roles would provide a framework for more effective communication and collaboration among the partners.

a *Structure evaluation:* adequacy in number and quality of materials (e.g., facilities, equipment, money, time), human resources (including participants), and organizational structure (4).

**Table 3:** Process Evaluation^a^ Questions and Potential Benefits for Healthy Weigh Phase 1, Tarrant County, Texas, 2003

**Process Question**	**Potential Benefits for Healthy Weigh Phase 1**
Were the design features of the program components effective? *Physical activity classes* Chair exercise Adult and adolescent female class Adult and adolescent male class School-aged youth *Nutrition classes* Adult/adolescent School-aged youth *Family meals* *Table talks* *Child care* *Screening* *Recruitment* Of participants Of volunteers *Retention* Of participants Of volunteers	Systematic identification, analysis, and documentation of best practices would accomplish the following: Help replicate and sustain the program Provide a clearer and more comprehensive understanding of what made recruitment and retention of participants effective Improve the experience of students in the program and the quality of the program through increased opportunities for students to build relationships with participants Promote investment of resources in staff building rather than managing staff turnover and shortages
Were community members and participants involved in designing and revising the program?	More active leadership roles for participants in the program may accomplish the following: Improve understanding of the relationship between participant involvement and program outcomes Promote participant feedback about existing program components and potential revisions Contribute to an earlier recognition of factors that facilitated and hindered participant involvement in leadership roles
Which other environmental factors (e.g., weather, community activities, guests, transportation) affect attendance and program implementation?	Clear understanding of all factors affecting participant attendance could result in better use of human and material resources needed for physical activity and nutrition classes, family meals, and food-bag preparation.
Which processes were in place to train and manage staff? Volunteer staff Paid staff	Training and management for volunteer and paid staff would be planned and implemented more proactively. Staff management could be evaluated based on commonly accepted human resources and volunteer administration standards.
Were language and cultural differences identified and addressed?	Proactive monitoring of language and cultural factors could result in early and accurate assessment of language skills of staff and participants and students' cultural competence skills. Investigators would be able to provide more timely teaching and mentoring to develop students' cultural competence skills.
Was the process for monitoring and evaluating the balance of needs among the needs of the participants, program, knowledge generation, and service learning effective (22)? How are the partnerships functioning?	Monitoring the balance of needs is an essential aspect of effective community-based participatory research partnerships. Establishing evaluation standards for the partnerships could be the basis for the following: Ongoing monitoring of the relationships Frequent acknowledgments of strengths and growth Rapid response to actual and potential problems
Were objectives related to service learning addressed?	Accurate documentation of best practices and strategies for promoting quality service learning experiences would provide data for a more comprehensive assessment of the value of service learning in this program.
How effective and efficient was the system for collecting and managing data to achieve comprehensive evaluation?	Efficient and effective data collection and management systems with requisite resource allocation makes comprehensive evaluation more likely.

a *Process evaluation:* how activities are carried out (4).

**Table 4:** Outcome Evaluation^a^ Questions and Potential Benefits for Healthy Weigh Phase 1, Tarrant County, Texas, 2003

**Outcome Question**	**Potential Benefits for Healthy Weigh Phase 1**
What program outcome information (other than that identified in the list of objectives in text) would contribute to meaningful understanding of Healthy Weigh Phase 1?	Broadening the scope of outcome evaluation to include more qualitative data could provide valuable insight into personal, family, and community behaviors that affect healthy eating, physical activity, and weight management. Behavioral, social, economic, and political change identified through quantitative and qualitative data could provide greater understanding about the short-term impact of Healthy Weigh Phase 1 on the following: Individual participants Families Community partner agencies Students Investigators
What other program outcome information would be important for determining the long-term impact of Healthy Weigh Phase 1?	Ongoing quantitative and qualitative evaluation of behavioral, social, economic, and political changes as a result of Healthy Weigh Phase 1 could provide better understanding of the long-term impact on participants and their families, community partners, students, and investigators.

a *Outcome evaluation:* the program's effects on participants (4).
