# Supplementary figures and images for: Healthy Weigh (El camino saludable) Phase 1: A Retrospective Critical Examination of Program Evaluation
Source: Prev Chronic Dis. 2006 Jun 15;3(3):A98. (PMC1656862)

Figure 1. Healthy Weigh/EI camino saludable, Phase 1/Fase 1 Logic Model

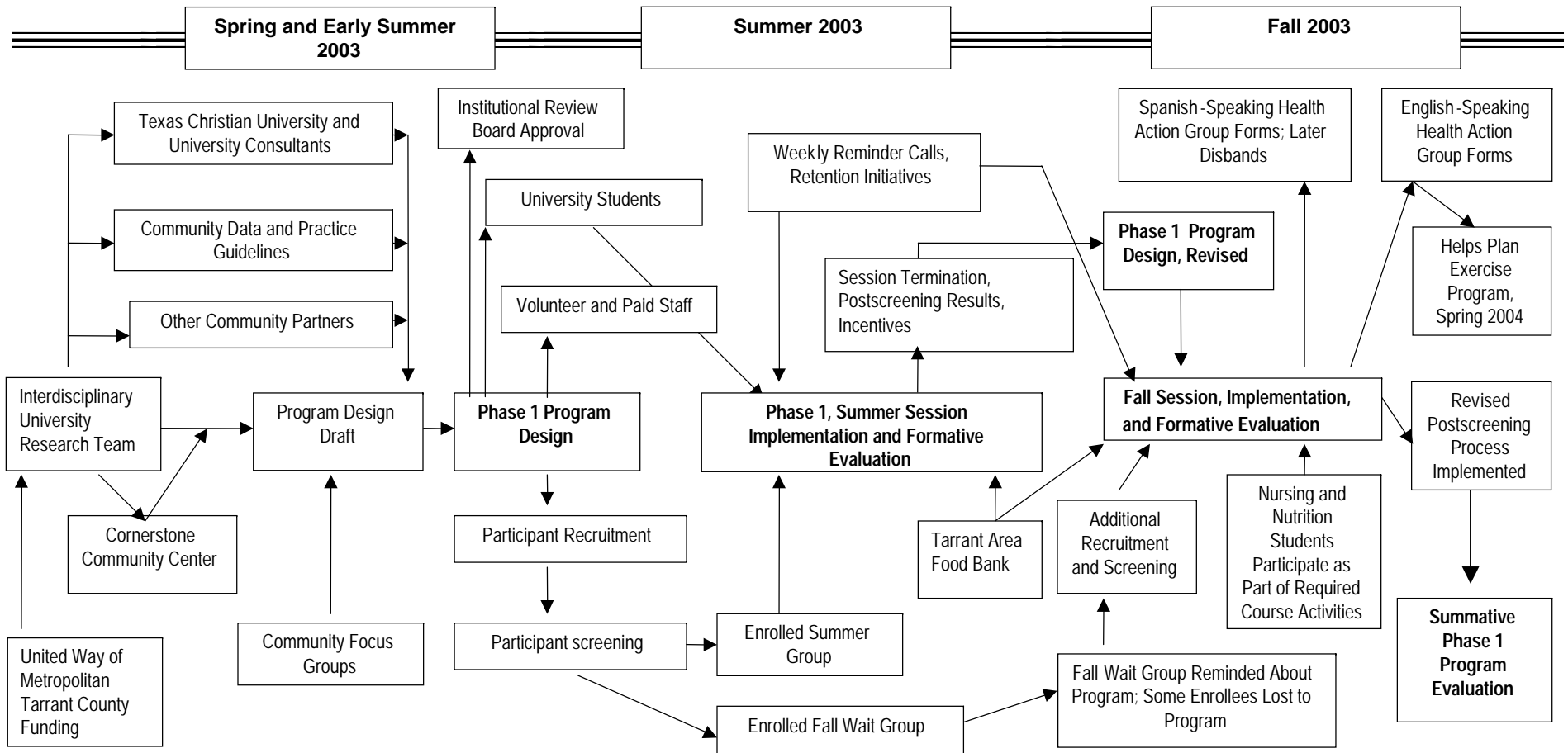

Supplement: Supplementary file 1 [file 05_0149_01.pdf]
